# Prevalence and Risk Assessment of Multiple Mycotoxins in Durum Wheat from Fields Under Different Agricultural Practices in Tunisia

**DOI:** 10.3390/toxins17080410

**Published:** 2025-08-14

**Authors:** Marwa Hassine, Khouloud Ben Hassouna, Salma Tissaoui, Mokhtar Baraket, Amine Slim, Olfa Ayed Slama, Hajer Slim Amara, Ahmed Al-Amiery, Noelia Pallarés, Houda Berrada, Samir Abbès, Jalila Ben Salah-Abbès

**Affiliations:** 1 Regional Field Crops Research Center of Beja, IRESA, Beja 9000, Tunisia; marwa.hassine1@gmail.com; 2 LR14AGR01, Laboratory of Genetics and Cereal Breeding, National Agronomic Institute of Tunisia, University of Carthage, Tunis 1082, Tunisia; amine_slim_inat@yahoo.fr (A.S.); olfayed@yahoo.fr (O.A.S.); amarahajer@yahoo.fr (H.S.A.); 3 Laboratory of Genetic, Biodiversity and Bio-Resources Valorisation, University of Monastir, Monastir 5000, Tunisia; khouloudhsn20@gmail.com (K.B.H.); abb_samir@yahoo.fr (S.A.); jalila.bensalah@yahoo.fr (J.B.S.-A.); 4 LR14AGR02, Laboratory of Bio-Aggressors and Integrated Pest Management in Agriculture, National Agronomic Institute of Tunisia, University of Carthage, Tunis 1082, Tunisia; salmatissaoui2@gmail.com; 5 National Research Institute for Rural Engineering, Water and Forests, University of Carthage, 17 Street Hédi Karray, BP No. 10, Ariana 2080, Tunisia; moktar.baraket@gmail.com; 6 National Gene Bank of Tunisia, Boulevard du Leader Yasser Arafat Z. I Charguia 1, Tunis 1080, Tunisia; 7 Al-Ayen Scientific Research Center, Al-Ayen Iraqi University, An Nasiriyah 64004, Thi Qar, Iraq; dr.ahmed1975@gmail.com; 8 Nutrition and Food Science Area, Preventive Medicine and Public Health, Food Science, Toxicology and Forensic Medicine Department, Faculty of Pharmacy, Universitat de València, 46100 Burjassot, Spain

**Keywords:** mycotoxins, durum wheat, soil tillage practices, Tunisia, UHPLC-MS/MS, risk assessment

## Abstract

Mycotoxin contamination in wheat, a staple food critical to human nutrition, poses significant public health concerns. This study investigated the natural occurrence of 17 mycotoxins in Tunisian durum wheat, assessed the influence of soil tillage practices on mycotoxin contamination, and performed an associated exposure risk assessment. A total of 167 wheat samples were randomly collected over two years (2021 and 2022) from fields managed under conventional tillage (CT) and no-tillage (NT) systems during both pre- and post-harvest periods. Mycotoxins were extracted using the QuEChERS method and quantified via UHPLC-MS/MS. The results demonstrated contamination by ZEN, DON, OTA, ENA1, ENB, and ENB1. Among regulated mycotoxins, OTA was the most prevalent, detected in 68 out of 167 samples with a mean concentration of 1.85 µg/kg. ZEN was the most abundant, detected in 65 samples with a mean concentration of 26.85 µg/kg, while DON was less frequently detected in 62 samples with a mean concentration of 0.68 µg/kg. Regarding emerging mycotoxins, ENB was the most prevalent and abundant, found in 51 samples with a mean concentration of 10.13 µg/kg; ENB1 and ENA1 were detected in 20 and 10 samples, with mean concentrations of 3.38 µg/kg and 1.69 µg/kg, respectively. Furthermore, mycotoxin concentrations varied according to agricultural practices. DON, ZEN, ENA1, ENB, and ENB1 showed higher frequencies and concentrations (ranging from 0.08 to 210.11 µg/kg) in samples collected during the 2021 pre-harvest period from NT fields. In contrast, OTA exhibited greater prevalence and higher concentrations (ranging from 2.33 to 9.78 µ/kg) in samples collected during the 2022 post-harvest period from CT fields. The Estimated Daily Intake (EDI) of mycotoxins by Tunisian adults was calculated based on contamination levels in raw durum wheat from fields under NT and CT practices, resulting in the following values (ng/kg bw/day), with the first value corresponding to NT samples and the second to CT samples: OTA (17.3; 20.8), ZEN (466.3; 194.0), DON (8.0; 7.56), ENA1 (4.30; 18.85), ENB (105.17; 121.08), and ENB1 (49.91; 40.91). Both the Margin of Exposure (MOE) values for OTA and the Hazard Quotients (HQ) for ZEN and DON exceeded established safety thresholds, indicating potential health risks for Tunisian adults. These findings highlight the urgent need to implement stricter mycotoxin regulations in Tunisia and enhance surveillance systems. Further research is warranted to elucidate the mechanisms by which soil tillage practices influence mycotoxin contamination and to develop targeted mitigation strategies to ensure food safety.

## 1. Introduction

Durum wheat (*Triticum turgidum* L. ssp. *Durum*) is the tenth most important cereal crop worldwide [1]. Durum wheat, which is known for its high protein content, golden color, and firm texture, serves as an important food source in certain regions [2], with a nutritional composition of 70% carbohydrates, 12–18% protein, 1.9% fat, 1.6% minerals, and 1.6% fiber [3]. It is grown in a broad range of environments, extending from Mexico, Russia, India, and North America to countries around the Mediterranean basin, including Tunisia [1]. In Tunisia, wheat stands as a cornerstone of agricultural and socioeconomic stability, deeply embedded in both dietary traditions and economic frameworks [4]. Wheat is Tunisia’s primary staple crop, accounting for 54% of the country’s cereal-growing area (about 1.5 million hectares) [5]. In fact, annual wheat cultivation covers over 700,000 hectares, with production concentrated in northern Tunisia, particularly in the region of Beja [6]. Tunisia is one of the leading countries in North Africa in terms of wheat consumption per capita, with an estimated annual intake of approximately 258 kg per person [5]. Tunisia, as a Mediterranean country characterized by a warm and humid climate, provides ideal conditions for the growth of toxigenic molds on grains [7]. These molds produce harmful substances called mycotoxins, which are mainly generated by fungi from the *Aspergillus*, *Alternaria*, *Claviceps*, *Fusarium*, and *Penicillium* genera [8]. Scientists have identified more than 500 types of mycotoxins worldwide. The most common ones found in wheat and food products include aflatoxins (AFs), ochratoxin A (OTA), zearalenone (ZEN), fumonisins (FMs), patulin (PAT), and trichothecenes (TRCs), including deoxynivalenol (DON) and T-2 toxin (T-2) [9]. The European commission [10] regulates these major mycotoxins through maximum allowable limits in food and feed. However, several Fusarium species (e.g., *F. acuminatum, F. avenaceum, F. oxysporum*, *F. poae*, *F. sporotrichioides*, *F. sambucinum*, and *F. tricinctum*) and Alternaria species (e.g., *A. alternata*, *A. tenuissima*, and *A. infectoria*) produce, respectively, unregulated mycotoxins, including enniatins (ENs) and alternariol (AOH), alternariol monomethyl ether (AME), tenuazonic acid (TeA), altenuene (ALT), tentoxin (TEN), and altertoxins (ATX-I, ATX-II, and ATX-III), which are considered emerging mycotoxins [11,12]. These mycotoxins are frequently detected in cereals and fruits; however, they remain unregulated due to the absence of standardized monitoring protocols and limited toxicological data [11,13]. Among the enniatin (EN) group, enniatin A (ENA), enniatin A1 (ENA1), enniatin B (ENB), and enniatin B1 (ENB1) are the most studied and commonly detected in cereals [14]. However, data on the occurrence and distribution of these emerging compounds remain scarce, particularly in developing countries, including those of North Africa, where surveillance programs are often lacking or insufficient.

Weather conditions such as prolonged humidity, unseasonal rainfall, or temperature extremes create optimal conditions for fungal growth and mycotoxin production [15]. However, these natural factors do not act alone; they interact with farming practices, especially soil tillage. Conventional tillage (CT), which involves plowing and turning over the soil, breaks up fungal networks and buries crop residues, potentially reducing the presence of mycotoxigenic fungi [16]. In contrast, no-tillage (NT) practices leave the soil undisturbed and preserve soil structure, moisture, and microbial biodiversity; they can also create favorable conditions for the survival and persistence of crop residues on the surface, including those capable of producing mycotoxins [17]. Moreover, tillage practices not only affect fungal load directly but also shape the composition and activity of soil microbial communities. A recent study has shown that the interaction between soil nutrients, rhizosphere metabolites, and microbes plays a key role in plant stress responses [18], which can indirectly influence fungal colonization and mycotoxin production. Therefore, understanding how different tillage systems modify the soil microbiome is essential for assessing mycotoxin contamination.

Wheat contamination by mycotoxins can occur at various stages along the agricultural supply chain, including before, during, and after harvest, particularly in wheat grains [19]. Moreover, multiple types of mycotoxins can contaminate wheat samples simultaneously [20]. In fact, it is estimated that mycotoxins affect 25% of the grains consumed worldwide, and this number could be even higher [21]. Mycotoxins represent a significant global public health threat due to their varied toxic effects on both humans and animals. These effects can range from acute poisoning to long-term health issues, including an increased risk of cancer, as well as cytotoxicity, hepatotoxicity, immunosuppression, genotoxicity, and nephrotoxicity [22]. Despite these risks, numerous studies across Europe, North America, and Asia have documented the occurrence of regulated mycotoxins such AFs, OTA, FBs, DON, and ZEN and emerging mycotoxins such as ENA, ENA1, ENB, and ENB1 in cereals, including durum wheat and wheat-based products [23,24]. Mycotoxins have also been detected in cereals and cereal-based products in Tunisia [7,25,26,27], including both regulated compounds such as AFB_1_, DON, and ZEN and emerging compounds like ENB and ENB1; however, the number of studies remains limited and does not provide a comprehensive overview of their occurrence across different regions and production conditions.

The detection and quantification of mycotoxins are primarily achieved through widely used methods such as Liquid Chromatography (LC) coupled with tandem mass spectrometry (MS/MS) and Liquid Chromatography (LC) coupled with a Fluorescence Detector (FD) [28]. Among these, Ultra-High-Performance Liquid Chromatography coupled with MS/MS (UHPLC-MS/MS) stands out for its high sensitivity and accuracy in detecting multiple mycotoxins, especially those lacking fluorophore structures [11]. Sample preparation typically involves an extraction step followed by a clean-up step to remove potential interferences. Among the various extraction approaches, the QuEChERS method—an acronym for Quick, Easy, Cheap, Effective, Rugged, and Safe—has emerged as one of the most widely adopted dispersive solid-phase extraction (d-SPE) techniques. Recent studies have adopted the QuEChERS method for the extraction of both regulated and emerging mycotoxins due to its simplicity and efficiency [22,29]. This approach offers a sample preparation procedure capable of simultaneously extracting multiple mycotoxins with minimal solvent use [29]. Due to their versatility and effectiveness in processing complex food matrices, QuEChERS-based methods are increasingly employed for routine monitoring of mycotoxin contamination in cereals such as wheat, barley, and maize [30]. The QuEChERS procedure generally involves two main steps. The first step consists of a partitioning process, where a salt solution and an organic solvent are used to separate the sample into inorganic and organic phases [30]. This step facilitates the extraction of analytes with varying polarities, as the combination of salts and solvents enhances analyte separation [31]. The second step includes a clean-up phase, during which sorbents such as C18 (Octadecyl silica) are used to remove non-polar interferences from the extract [32].

Despite the significant consumption of wheat in Tunisia, which is used in various forms such as unprocessed grains, flours, and processed products like bread, besissa, breakfast cereals, couscous, pasta, and cakes [7], the natural occurrence of mycotoxins in durum wheat remains inadequately investigated. To date, most studies have concentrated primarily on a limited number of regulated mycotoxins, while emerging mycotoxins have received limited scientific attention and remain insufficiently characterized. Furthermore, no recent comprehensive research has been conducted on the co-occurrence of both regulated and emerging mycotoxins in locally produced cereals, including durum wheat. This represents a significant knowledge gap that limits our ability to accurately assess mycotoxin exposure and to apply effective risk control measures. While agricultural practices are widely acknowledged to influence the occurrence of mycotoxin contamination, their impact has yet to be investigated in Tunisian studies. Furthermore, there is a lack of research on the risk assessment of mycotoxin exposure in Tunisia, limiting the understanding and management of related health risks. The scarcity of recent and comprehensive data on both regulated and emerging mycotoxins in Tunisian cereals including durum wheat represents a significant knowledge gap. This is particularly relevant in light of changing climatic conditions and agricultural practices, which may influence fungal contamination and toxin production. Therefore, generating updated data on the occurrence and co-occurrence of multiple mycotoxins in durum wheat is essential to improve risk assessment and support the development of appropriate national monitoring and control strategies.

To the best of our knowledge, no prior Tunisian studies have systematically investigated the occurrence of multi-mycotoxins in wheat from fields under different soil tillage practices, followed by comprehensive exposure assessments and risk characterization.Therefore, the present study aimed to evaluate the natural prevalence of multiple mycotoxins—including OTA, ENA1, ENB, ENB1, ZEN, FUS-X, NEO, HT-2 toxin HT2, T-T2, NIV, DON, AcDON, 3ADON, FB1, FB2, and CIT—in durum wheat samples from fields with NT practices and those employing CT practices during the pre- and post-harvest periods of 2021 and 2022 to study the effect of agriculture practices on the presence of mycotoxins in wheat, as well as to assess Tunisian adult dietary exposure levels to the detected mycotoxins, followed by risk characterization. To the best of our knowledge, no prior Tunisian studies have systematically investigated the occurrence of multiple mycotoxins in wheat from fields under different soil tillage practices, followed by comprehensive exposure assessments and risk characterization.

## 2. Results and Discussion

### 2.1. Occurrence of Mycotoxins in Tunisian Durum Wheat

A total of 167 raw durum wheat samples from Tunisia were analyzed using UHPLC-MS/MS to evaluate the natural occurrence of 17 mycotoxins. This analysis included regulated mycotoxins (ZEN, CIT, OTA, DON, HT-2, T-2, FUM B1, FUM B2, and NIV). Additionally, it assessed other mycotoxins not covered by EU legislation, which are relevant for food safety considerations, including FUS-X15, AcDON, 3AcDON, and NEO. The study also highlighted emerging *Fusarium* mycotoxins, specifically ENA, ENA1, ENB, and ENB1. Table 1 displays the occurrence, mean concentration, and concentration range (minimum and maximum values) of the detected mycotoxins in positive durum wheat samples. A sample was considered positive when its concentration exceeded the limit of detection (LOD).

The results indicated contamination of wheat samples by ZEN, OTA, DON, ENA1, ENB, and ENB1. OTA was the most frequently detected mycotoxin, found in 40.71% of samples, followed by ZEN at 38.92%, DON at 37.12%, ENB at 30.53%, ENB1 at 11.98%, and ENA1 at 5.98%. OTA was detected at concentrations ranging from 1.02 to 11.45 µg/kg, and among the positive samples, 23 samples (33.82%) exceeded the EU-authorized maximum concentration of 5 µg/kg for OTA in unprocessed cereals [33]. OTA was also detected in other wheat samples from Tunisia. Similarly to our findings, Ben Hassouna et al. [26] reported its presence in 11.11% of analyzed samples at high concentrations ranging from 2.47 to 9.13 µg/kg, and 10% of these samples exceeded the authorized concentration limit of 5 µg/kg. The presence of OTA is not limited to Tunisia; it has also been detected in wheat samples from other North African countries. For instance, Riba et al. [34] found OTA in Algerian wheat grains at concentrations ranging from 0.21 to 27.31 µg/kg, which were higher than those observed in our study. In contrast to our findings, Hathout et al. [35] reported lower concentrations of OTA in wheat samples from Egypt, with levels ranging from the LOQ to 1.70 µg/kg, none of which exceeded EU-authorized limits. Furthermore, OTA has been detected in other cereal types from Tunisia. For example, Houissa et al. [36] reported higher concentrations of OTA in Tunisian pearl millet, ranging from 121 to 1480 µg/kg. In contrast, Juan et al. [27] found OTA in only two Tunisian barley samples at low concentrations of 2.9 and 3 µg/kg. The elevated levels of OTA observed in our study could be associated with Tunisia’s Mediterranean climate, which may promote the growth of *Aspergillus* and *Penicillium* species that produce OTA [7]. In fact, in a prior study, a highly OTA-producing strain of *Aspergillus niger* was found to contaminate wheat, barley, and maize in Tunisia [37].

ZEN was detected at concentrations ranging from 10.14 to 255.41 µg/kg, with a mean value of 26.85 µg/kg. Among the ZEN-contaminated samples, 21 (32.30%) exceeded the maximum level of 100 µg/kg established by the European Union for ZEN in unprocessed cereals other than maize [33]. The significant levels found in Tunisian wheat reflect broader trends observed across Tunisia and other North African countries, where ZEN has been detected in both wheat and other cereal types. For instance, Zaied et al. [38] detected ZEN in 80% of durum wheat samples from Tunisia, reporting high concentrations with a mean of 58 µg/kg. More recently, Jedidi et al. [39] identified ZEN in Tunisian durum wheat at concentrations ranging from 21.5 to 32.4 µg/kg, which are lower than the levels found in our study. In Algeria, Mahdjoubi et al. [40] reported ZEN concentrations in wheat ranging from 16.6 to 47.2 µg/kg, also lower than our findings. In contrast to our findings, El-Desouky et al. [41] found ZEN in wheat from Egypt at low concentrations between 0.7 and 1.77 µg/kg. DON was detected in concentrations ranging from 0.01 to 9.11 µg/kg. All positive samples remained well below the maximum limit of 1750 µg/kg set by the European Union for DON in unprocessed durum wheat grains [33]. However, one previous study reported higher levels in Tunisian wheat, with concentrations ranging between 12.8 and 30.5 µg/kg [42]. Additionally, Oueslati et al. [43] analyzed DON in bread made from wheat and detected concentrations ranging from 1 to 25.2 µg/kg. In contrast, Juan et al. [27] examined Tunisian barley and reported lower concentrations of DON, ranging from 1.7 to 6.1 µg/kg. Regarding the emerging *Fusarium* group, ENA1, ENB, and ENB1 were detected in raw wheat samples in the current study at high levels. ENB emerged as the most prevalent compound with a mean concentration of 10.13 µg/kg, followed by ENB1 (3.38 µg/kg) and ENA1 (1.69 µg/kg), establishing a clear descending order of abundance: ENB > ENB1 > ENA1. Recently, Aloui et al. [25] observed the same contamination ratio (ENB > ENB1 > ENA1) in durum wheat from Beja, the same region as in our study, but a higher mean ENB concentration of 350.7 µg/kg. In contrast to our findings, Oueslati et al. [44] observed a different contamination ratio of ENs in raw durum wheat from Tunisia, with the order being ENA1 > ENB > ENB1. They reported a mean concentration of 90,000 µg/kg for ENA1, 75,000 µg/kg for ENB, and 48,000 µg/kg for ENB1, all of which are higher than the concentrations found in the current study. In another study by Oueslati et al. [45], the presence of ENs in raw Tunisian wheat destined for children’s consumption was highlighted. However, they only identified ENA1 and ENB1, each in a single sample, at very low concentrations (ENA1: 0.011 µg/kg and ENB1: 0.006 µg/kg), much lower than those observed in our study. In Tunisian durum wheat from the same region as in our study (Beja), Chakroun et al. [46] also identified ENs, but only ENA and ENB1, with high concentrations ranging from 259,000 to 931,000 µg/kg for ENA and from 25,100 to 43,000 µg/kg for ENB1, respectively. ENs were also detected in other cereal types from Tunisia. For instance, Juan et al. [27] also investigated the presence of ENs in barley and detected all four mycotoxins (ENA, ENA1, ENB, and ENB1). They found the same order of mycotoxins (ENB > ENB1 > ENA1) based on mean concentrations, reporting levels of 18.5, 14.7, and 10.9 µg/kg, respectively, which are higher than our findings. More recently, Juan et al. [47] investigated the presence of ENA, ENA1, ENB, and ENB1 in silage cereals from the same region as in our study, but they only detected ENB at a very low average concentration of 0.063 µg/kg.

The accumulation of mycotoxins from *Aspergillus*, *Penicillium*, and *Fusarium* in Tunisian wheat samples highlights the environmental factors that contribute to fungal contamination. In fact, the Mediterranean climate of Tunisia, characterized by warm temperatures and intermittent humidity, creates an environment conducive to fungal growth and mycotoxin production [7]. On top of this, a critical factor in regulating fungal proliferation and mycotoxin biosynthesis is water activity (aw). As indicated in Table 2, all analyzed wheat samples had aw values below 0.7 (ranging from 0.59 to 0.7), which is typically considered insufficient for supporting the metabolic processes of water-dependent fungi [25]. Despite these low aw levels, mycotoxigenic fungal activity was still detected. This suggests that mycotoxins can be produced even when water activity is below 0.7. For instance, Estrada-Bahena et al. [48] found that the optimal water activity values for the highest levels of OTA in dry cherries and green coffee beans were 0.628 and 0.743, respectively. Moisture content also significantly influences mycotoxin production. Generally, the production of mycotoxins is favored when relative humidity is between 88% and 95% [49]. However, as shown in Table 2, the moisture content in all analyzed samples did not exceed 16%. This indicates that while the environmental conditions may not seem ideal for mycotoxin production, other factors are probably involved.

### 2.2. Distribution of Mycotoxins in Durum Wheat Under Varied Conditions in 2021 and 2022

Diverse mycotoxin distribution profiles were identified in the wheat samples analyzed from the pre-harvest and post-harvest seasons of 2021, cultivated in NT fields, as well as from the corresponding season in 2022, cultivated in CT fields (see Table 3 and Table 4). OTA, ZEN, DON, ENB1, and ENA1 were detected in samples collected during both the pre-harvest and post-harvest seasons of 2021 and 2022. However, ENB was not detected in the post-harvest samples from 2021.

In 2021, samples collected during the pre-harvest season showed higher levels of DON (0.08–9.11 µg/kg), ENA1 (5.52–10.74 µg/kg), and ENB (8.81–247 µg/kg). This increase can be linked to the greater susceptibility of wheat to *Fusarium* infection during the anthesis stage, a crucial period in its reproductive development when mycotoxins are predominantly synthesized by *Fusarium* species in the fields [50]. During that year, the Beja region experienced substantial pre-harvest rainfall, with 62 mm in May and 32 mm in June. These conditions significantly elevated the risk of *Fusarium* mycotoxin accumulation in wheat, a phenomenon further exacerbated by concurrent factors such as elevated air temperature and high relative humidity [51]. Conversely, in 2022, DON and ENs were predominantly detected in post-harvest samples, with concentrations ranging from 0.05 to 5.14 µg/kg for DON, from 2.10 to 20.78 µg/kg for ENA1, from 8.20 to 63.3 µg/kg for ENB, and from 3.29 to 60.42 µg/kg for ENB1.

High concentrations of DON, ENB, and ENA1 were observed in pre-harvest wheat samples from 2021 compared to those from 2022. This discrepancy can be attributed to the climatic conditions in those two years, particularly rainfall, which may have intensified the contamination of *Fusarium* mycotoxins in wheat during the pre-harvest period [52].

In fact, in Tunisia, 2021 experienced significantly more rainfall than 2022, which faced an unexpected drought [53]. Prolonged rainy weather during critical growth stages, from March to the end of May, resulted in an average rainfall of 47 mm in 2021, compared to just 7 mm in 2022, which significantly increased moisture levels, thereby favoring the development of fungi and the production of mycotoxins, particularly DON and ENs, in the field [51]. Moreover, agricultural practices played a significant role; in 2021, NT methods were employed, which can greatly influence the presence of DON, ENA1, ENB, and ENB1 in wheat. Similar findings obtained by Blandino et al. [54] indicated that DON contamination in cereals is more severe when minimum or NT practices are applied. ZEN was detected in 2021 at concentrations ranging from 10.14 to 210.11 µg/kg in pre-harvest samples and from 12.11 to 255.41 µg/kg in post-harvest samples. In 2022, the concentrations were lower in pre-harvest samples, ranging from 44.56 to 122.56 µg/kg, while concentrations in post-harvest samples varied from 10.71 to 211.25 µg/kg. ZEN, a mycotoxin primarily produced by *Fusarium* fungi in the field before harvest, was frequently and abundantly detected in post-harvest wheat samples from both 2021 and 2022. This prevalence can be attributed to several environmental factors, including high moisture levels and poor ventilation during harvest, as well as conditions present during subsequent storage [55]. Additionally, the use of non-airtight storage materials, such as jute or woven polypropylene sacks, after harvest may further contribute to contamination [56].

The results of this study also suggest that the presence of hotspots during the transport of wheat post-harvest promotes *Fusarium* proliferation, leading to increased levels of ZEN in the stored grains. The variation in the prevalence of ZEN during the pre-harvest seasons of 2021 and 2022 can also be attributed to agricultural practices. In 2021, ZEN was frequently detected at high concentrations in samples from fields characterized by NT practices, primarily due to the extended presence of organic matter and moist conditions that promote fungal growth [57]. Additionally, as noted by Mielniczuk and Skwaryło-Bednarz [16], the agricultural methods employed prior to crop production can significantly impact the subsequent contamination of small-grain cereals with *Fusarium* mycotoxins. Moreover, so much importance has been given to tillage practices that some authors have prioritized land preparation such as tillage, cover crop, and crop rotation when describing the fundamental GAP to control mycotoxin contamination in cereals [58].

OTA was detected in both pre-harvest and post-harvest samples from 2021 and 2022; however, it was significantly more abundant in post-harvest samples during both years, with concentrations ranging from 1.23 to 11.45 µg/kg in 2021 and from 2.33 to 9.78 µg/kg in 2022. In fact, species of the genus *Aspergillus and pencelium,* producers of OTA, are generally considered storage fungi as they usually contaminate products in the post-harvest stages [59], although they are sometimes also present in the field [60]. Poor post-harvest practices, such as insufficient drying or exposure to moisture and poor storage conditions, significantly increase contamination risks. In Tunisia, farmers often use non-airtight storage materials, such as jute or woven polypropylene sacks. While these sacks are commonly used, their permeable nature allows for unrestricted airflow, creating an environment conducive to the proliferation of fungal spores and the production of mycotoxins [56]. In addition, insufficient drying of crops after harvest and before storage exacerbates fungal growth and mycotoxin accumulation in stored wheat [58]. The presence of OTA in pre-harvest wheat samples can be attributed to the climatic conditions prevalent during this period. *Penicillium verrucosum*, the primary OTA-producing fungus, exhibits optimal growth at temperatures around 20–25 °C [59]. This range aligns closely with the average temperatures recorded in our study region, Beja (approximately 24–26 °C), during the weeks preceding wheat harvest, creating a favorable environment for fungal proliferation and subsequent OTA biosynthesis.

OTA was found to be more frequent in pre-harvest samples from fields under CT practices (2021), exhibiting higher levels of contamination compared to samples from fields that utilized NT practices. Specifically, OTA was detected in 13 samples in 2021 at concentrations ranging from 1.56 to 9.55 µg/kg, while in 2022, it was found in 20 samples at concentrations between 1.02 and 8.56 µg/kg. This increase in contamination can be attributed to the greater soil disturbance associated with conventional tillage.

In fact, the improved aeration and warming of the soil under CT create elevated moisture levels that are conducive to fungal proliferation [17].

Tillage practices are crucial before planting, as they help mitigate the risk of *Fusarium* and *Aspergillus* infection by reducing the persistence of their spores in crop residues, particularly when crop stover remains on the soil surface [17]. However, our study found that tillage practices did not have a significant impact on the incidence of OTA. This highlights the complexity of the relationship between tillage and mycotoxin contamination, as the impact of reduced tillage on the presence of contaminants in wheat grain remains a topic of ongoing debate [61].

Given this uncertainty, it is important to weigh the advantages of different agricultural practices in durum wheat production against their potential limitations. While conventional tillage may reduce fungal residues, it can harm soil structure and increase erosion. In contrast, no-tillage practices preserve soil health and microbial diversity but may favor the persistence of mycotoxigenic fungi under certain conditions. These contrasting outcomes underline the need for integrated strategies that ensure both crop protection and soil sustainability. As a complementary approach, microbial-based biocontrol solutions are showing promising potential. Liu et al. [62] demonstrated that synthetic Bacillus consortia can enhance plant growth and resilience through cooperative interactions in the rhizosphere. These beneficial microbes may help suppress pathogenic fungi and reduce mycotoxin contamination. Incorporating such microbial tools into cereal production systems, adapted to specific soil and tillage conditions, could strengthen existing control measures and contribute to safer, more sustainable agriculture.

### 2.3. Co-Occurence of Mycotoxins in Analyzed Durum Wheat

The current study evaluated the natural co-occurrence of mycotoxins in the analyzed wheat samples (Table 5). The results showed significant contamination by multiple mycotoxins, with up to five different types detected in a single sample. The identified mycotoxins included regulated varieties such as ZEN + OTA, DON + OTA, and ZEN + OTA + DON. Additionally, emerging mycotoxins were found both individually, such as the combination of ENB + ENB1, and in combination with regulated mycotoxins, such as ZEN + OTA + ENA1 + ENB + ENB1 and DON + ENB1.

The natural co-occurrence of mycotoxins in cereals has also been reported in various studies conducted in Tunisia. For instance, Juan et al. [27] reported that barley samples were contaminated with up to four mycotoxins, including regulated ones like OTA, ZEN, and DON, as well as ENs, with dual co-occurrence being the most common finding. Furthermore, Ben Hassouna et al. [26] observed the co-presence of AFG2 and OTA in 17.30% of positive wheat samples from Béja, the same region as that covered by our study. In line with our findings, Oueslati et al. [43] identified the simultaneous presence of DON, ENB, and ENB1 in 65% of Tunisian cereal biscuits.

In 2021, wheat samples from fields with NT practices showed that two-mycotoxin combinations were the most prevalent, accounting for 27% of positive samples. The co-occurrence of three and four mycotoxins was observed in 10% and 7% of positive samples, respectively. The co-occurrence of five mycotoxins was less frequent, representing only 5% of positive samples. In contrast, data from 2022 revealed that samples from fields employing CT exhibited a distinct trend, showing a higher prevalence of both two- and three-mycotoxin combinations compared to the data from 2021. Two-mycotoxin co-occurrence was the most prevalent, accounting for 28% of positive samples that year. This was followed by the co-occurrence of three mycotoxins. The co-occurrence of four and five mycotoxins was less frequent, at 5% and 3% of positive samples, respectively. The observed increase in two- and three-mycotoxin combinations in wheat samples from fields under CT practices in 2022, compared to those from fields with NT practices in 2021, may be attributed to altered fungal ecological dynamics. In fact, CT practices disrupt soil structure and residue distribution, potentially creating heterogeneous microenvironments that favor the coexistence of multiple fungal species with diverse mycotoxin profiles [63]. These practices may also enhance spore dispersal and modify substrate availability, facilitating simultaneous colonization by toxigenic fungi such as *Aspergillus*, *Fusarium*, and *Penicillium* species [64]. Furthermore, the physical damage inflicted on crops by tillage could compromise plant defenses, increasing susceptibility to poly-infection scenarios involving multiple fungi [64].

### 2.4. Risk Assessment

#### 2.4.1. Dietary Exposure to Mycotoxins

Daily, individuals are exposed to mycotoxins by consuming contaminated wheat or wheat products. Dietary exposure assessment, an integral part of risk assessment methodology, combines mycotoxin concentrations in food with population consumption patterns. This approach provides critical insights for effective mycotoxin control [65]. In this context, we assessed the Estimated Daily Intake (EDI) of each mycotoxin detected in the survey to evaluate the associated risks of mycotoxin consumption through wheat among Tunisian adult consumers weighing 70 kg. In Tunisia, the average wheat consumption is estimated at 716.66 g/day [6].

Table 6 summarizes the mean concentrations and EDI values for mycotoxins detected in Tunisian wheat samples collected in 2021 from fields with NT practices and those collected in 2022 from fields under CT practices. The EDIs of mycotoxins from Tunisian wheat grains demonstrated year-to-year variations.

In 2021, the EDI values were 17.29 ng/kg bw/day for OTA, 466.31 ng/kg bw/day for ZEN, 8.00 ng/kg bw/day for DON, 4.30 ng/kg bw/day for ENA1, 105.17 ng/kg bw/day for ENB, and 49.91 ng/kg bw/day for ENB1. For the 2022 samples, the corresponding EDI values were 20.08 ng/kg bw/day for OTA, 194.74 ng/kg bw/day for ZEN, 7.56 ng/kg bw/day for DON, 18.85 ng/kg bw/day for ENA1, 121.08 ng/kg bw/day for ENB, and 40.92 ng/kg bw/day for ENB1.

The EDI of DON from wheat collected in 2021 from fields with NT practices, as well as from CT fields in 2022, did not exceed the TDI for DON, which is set at 1000 ng/kg bw/day by the EFSA [66]. In contrast, the EDI of ZEN from wheat collected in 2021 from fields with NT practices exceeded the TDI of 250 ng/kg bw/day established by the EFSA [67]. However, ZEN exposure from wheat collected in 2022 from CT fields remained below this threshold. In previous studies available in the literature, Juan et al. [27] determined the EDI for ZEN and DON under upper-bound (UB) and lower-bound (LB) scenarios based on the consumption of Tunisian raw barley. Their findings revealed that the EDIs were significantly lower than those reported in our study for the same mycotoxins. Ben Hassouna et al. [26] assessed the EDI of OTA through the consumption of Tunisian raw durum wheat. They reported an EDI of 51.685 ng/kg bw/day for OTA, which is higher than the EDIs found in our current study. In contrast, Juan et al. [27] found an EDI of OTA equal to 0.09 and 0.02 ng/kg bw/day for the UB and LB scenarios, respectively, which are lower than our findings. Furthermore, the EDIs of emerging mycotoxins, specifically ENs, have been evaluated in other Tunisian studies. Aloui et al. [25] determined the EDIs of ENA1, ENB, and ENB1 through the consumption of durum wheat collected in 2020 and 2021 from Béja, the same region as in our study. In samples from 2021, the EDIs for ENA, ENB, and ENB1 were found to be 9.2, 786.6, and 102.4 ng/kg bw/day, respectively, which were higher than our findings. These differences can be attributed to variations in agricultural practices in the fields from which the samples were collected, as well as the months of collection and the analysis methods used.

While many studies have documented the contamination of Tunisian cereals, particularly wheat, with various mycotoxins [7], a significant knowledge gap persists regarding population-level exposure. Current data on dietary intake assessments and human exposure quantification through cereal consumption are notably scarce, limiting comprehensive understanding of public health implications and hindering focused risk management strategies in Tunisia. Due to the absence of wheat consumption data for different age groups in Tunisia, we were only able to estimate exposure for adult consumers.

#### 2.4.2. Risk Characterization

##### Risk Characterization by the MOE Approach

The health risk characterization of OTA exposure through durum wheat consumption among Tunisian adults was conducted via the MOE method. This approach is specifically designed to quantify potential risks associated with dietary intake of carcinogenic and genotoxic contaminants [68], enabling a science-based evaluation of OTA-related public health concerns in the context of Tunisia’s staple food consumption patterns. When the value of the MOE is greater than 10,000, it is assumed that there is a low risk to public health. According to Table 4, the MOE for OTA in our study was found to be 838.63 for wheat samples cultivated under NT practices in 2021 and 722.11 for those cultivated under CT practices in 2022. Both values are significantly below 10,000, indicating potential health risks associated with the consumption of OTA-contaminated wheat. In contrast to our results, De Sá et al. [69] reported an MOE for OTA in cereals from Portugal that exceeded 10,000, indicating no significant risk to human health. Contrary to our results, in Lebanon, which shares a comparable Mediterranean climate with Tunisia, Hoteit et al. [70] calculated the MOE for various types of cereals and found that all values were above 10,000, suggesting no risk to human health. Our findings align with those from Côte d’Ivoire, where the MOE values for OTA exposure through the consumption of contaminated rice and maize were below 10,000. This raises health concerns, as such exposure could potentially lead to neoplastic effects [71].

The lower MOE for OTA in CT samples suggests that the tillage method influences OTA levels, and further research is required to confirm causal relationships. Based on the existing literature, there is a notable absence of Tunisian studies specifically focused on calculating the MOE for OTA and assessing the associated risks from OTA ingestion. OTA is a highly toxic contaminant known for its potent carcinogenic properties. Its presence poses significant health risks, particularly through the consumption of durum wheat. Therefore, addressing the risk associated with the consumption of OTA in durum wheat in Tunisia should be regarded as a top priority for effective risk management strategies.

##### Risk Characterization by the HQ Approach

The health risk assessment of exposure to ZEN and DON through the consumption of durum wheat in Tunisia was conducted using the HQ approach. This approach assesses potential risks by comparing population-level dietary exposure estimates to the tolerable daily intake (TDI) values for ZEN and DON. These TDI values represent safety thresholds derived from animal studies and human risk assessments [72]. An HQ value below one typically indicates low risk for consumers.

Based on the results of mycotoxin exposure, the HQ values for ZEN and DON are presented in Table 4. For ZEN, the HQ value was 1.87 for wheat samples cultivated under NT practices in 2021, while the HQ for samples grown using CT practices in 2022 was 0.80. The HQ values for wheat samples revealed significant variations between years and farming methods. In 2021, the risk levels exceeded safety thresholds (HQ > 1), indicating a concerning level of risk, whereas in 2022, the HQ fell below the danger level (HQ < 1). Notably, NT farming practices were associated with more than double the risk of ZEN contamination compared to CT practices. Globally, there is a significant lack of data concerning the risk characterization of ZEN in cereals. In contrast to our results, a study by Palma et al. [73] reported HQ values of 7.7 and 4.3 for the age groups of 2–5 and 6–13 years, respectively, indicating a potential risk to consumers in Chile following ZEN exposure. In Portugal, safety concerns related to ZEN consumption through various cereal products were identified, with all determined HQ values exceeding 1 [69].

For DON, the HQ values were notably low: 0.008 for wheat samples with NT practices and 0.007 for those with CT practices. In both cases, the HQ values were far below 1, indicating no significant health risk from DON in wheat, regardless of farming method. Currently, there are no studies conducted in Tunisia, and there is a notable lack of research worldwide concerning the risk characterization of DON in cereals. In comparison to a Lebanese study, the research of Hoteit et al. [70] reported an HQ for DON of 0.0514, which is significantly below the threshold of 1. This indicates that there are no substantial risks to the Lebanese population of DON exposure through cereal consumption.

The characterization of risk has become unachievable for ENs because there are no TDIs set by the Joint FAO/WHO Expert Committee on Food Additives (JECFA) or the European Food Safety Authority (EFSA). This lack of guidelines makes it difficult to assess their possible health effects from wheat consumption in Tunisia. The risk characterization conducted in our study has several limitations regarding mycotoxin exposure evaluation. These include the uneven distribution of mycotoxins in wheat, the possibility of exposure through routes other than ingestion, and the occurrence of masked mycotoxins [74].

The findings of this study highlight a significant public health concern regarding Tunisian adults exposed to mycotoxins, particularly ZEN (detected in wheat samples from NT fields) and OTA, through the consumption of contaminated wheat. This situation is particularly significant considering the wide range of potential mycotoxin sources linked to the high wheat consumption among the Tunisian population, along with the simultaneous presence of multiple mycotoxins in one sample, up to five mycotoxins in this study.

While analytical data suggest that mycotoxins such as DON and, to a lesser extent, ZEN, particularly those detected in wheat samples from CT fields, do not present an immediate health risk for adult consumers in Tunisia, concerns persist regarding long-term exposure. Regular consumption of wheat-based products contaminated with mycotoxins can result in adverse health outcomes over time, emphasizing the need to acknowledge the associated risks of consuming durum wheat in Tunisia. To effectively address this issue, prioritizing risk management frameworks is vital, which should encompass implementing monitoring programs to track mycotoxin levels, educating farmers on control strategies, optimizing post-harvest storage practices, adopting sustainable tillage methods, and promoting resistant crop varieties.

## 3. Conclusions

The analytical method using UHPLC-MS/MS employed in the current study analyzed 17 mycotoxins in durum wheat grown in fields with CT and NT practices over two successive years (2021 and 2022), during the pre- and post-harvest periods in Tunisia. Wheat samples were found to be contaminated with OTA, ZEN, DON, ENA1, ENB, and ENB1. The most prevalent mycotoxin was OTA, while ENA1 was the least frequently detected. Notably, 33.82% of positive samples contained concentrations exceeding the maximum authorized limit for OTA (5 µg/kg) in unprocessed cereals. This study revealed high mycotoxin levels, reaching 384.20 µg/kg for ENB. Different levels and frequencies of mycotoxins were observed in samples collected from NT fields compared to those from CT fields during both the pre-harvest and post-harvest periods. The co-occurrence of mycotoxins in wheat has also been documented. Toxicological assessments, including EDI, MOE, and HQ, indicate that Tunisian adults are exposed to various analyzed mycotoxins due to high consumption of durum wheat, posing potential health risks. Although the levels of individual mycotoxins in Tunisian wheat may not currently exceed critical thresholds for certain mycotoxins, the cumulative risk of exposure to multiple toxins and the country’s dietary dependence on wheat necessitate a systematic public health response. Implementing integrated surveillance, promoting agricultural best practices, and raising consumer awareness will be essential in mitigating long-term health consequences. Furthermore, it is imperative to conduct additional studies in Tunisia that explore different soil tillage practices for wheat cultivation. Those studies may contribute to determining correlations with mycotoxin prevalence, enabling the identification of optimal cultivation methods to minimize mycotoxin production. Additionally, it is vital to establish robust regulatory frameworks for mycotoxin limits in Tunisian food, especially in wheat, to safeguard consumer health.

## 4. Materials and Methods

### 4.1. Study Region and Sampling Procedures for Durum Wheat

Beja Governorate, a major wheat-producing region located in northwestern Tunisia (geographic coordinates: 36°43′32.30′′ N latitude, 9°10′54.08′′ E longitude; altitude: 222 m above sea level), was chosen as the study region in the current research. In 2021 and 2022, 167 durum wheat samples were collected. In 2021, a total of 73 samples were collected from fields with no-tillage (NT) practices, comprising 37 samples from the pre-harvest phase (January–April) and 36 samples from the post-harvest phase (July–September). In 2022, a total of 94 samples were collected from fields using conventional tillage (CT) practices, during the same periods as in 2021, consisting of 49 samples from the pre-harvest phase and 45 samples from the post-harvest phase. All samples were processed according to Regulation EC 401/2006 guidelines [10], homogenized, milled into 100 g aliquots, stored in sterile polyethylene bags, and preserved at −20 °C prior to mycotoxin analysis. The water activity (aw) of wheat samples was measured using an AquaLab4TE meter (Decagon Devices Inc., Pullman, WA, USA). Moisture content was determined according to the ISO 712:2009 standard [10], where samples were homogenized into small particles (1.7 mm diameter) to ensure uniform drying. An isothermal oven (Chopin, France) was employed for controlled drying, followed by storage in desiccators containing dehydration agents to maintain stable humidity conditions during analysis.

### 4.2. Reagents and Standards

Analytical standards for OTA, ENA, ENA1, ENB, ENB1, ZEN, FUS-X, NEO, HT2, NIV, DON, AcDON, 3ADON, FB1, FB2, and CIT were sourced from R-Biopharm AG, France, with stock solutions stored at −20 °C in darkness. Working solutions were prepared by diluting stocks in the mobile phase (water/acetic acid, 99.5:0.5, *v*:*v*) and refrigerated at +4 °C until UHPLC-MS/MS analysis. Solvents, including LC-grade acetonitrile and acetic acid, were purchased from Fisher Chemical (Loughborough, UK), and LC-MS/MS-grade isopropanol was purchased from Biosolve (Dieuze, France). A QuEChERS EN 15662 kit purchased from Phenomenex (Torrance, CA, USA) was used for mycotoxin extraction, and ultrapure water was generated via a Milli-Q system (Merck Millipore, Germany).

### 4.3. Extraction Procedure

Mycotoxin extraction was conducted using a QuEChERS (quick, easy, cheap, effective, rugged, and safe) method adapted from a study by Aloui et al. [75]. Briefly, 5.0 g of homogenized wheat powder (weighed to ±0.0001 g precision using an analytical balance) was transferred into a 50 mL centrifuge tube, followed by the addition of 20 mL of extraction solvent (acetonitrile/water/acetic acid, 49.5:49.5:1, *v*:*v*:*v*). The mixture was vigorously shaken for 15 min using a multi-axis vortex agitator (PTR-35, Grant Bio) to ensure thorough homogenization. Subsequently, a QuEChERS EN 15662 extraction kit (Torrance, CA, USA) was introduced to the sample for cleanup. After 10 min of shaking, the sample underwent centrifugation at 9000 rpm for 10 min to separate phases. A 200 µL aliquot of the supernatant was transferred to a vial, mixed with 600 µL of water/acetic acid (99.5:0.5, *v*/*v*), and filtered through a 0.45 μm PTFE syringe filter to remove particulates. Prior to UHPLC-MS/MS analysis, 400 µL of the filtrate was spiked with 100 µL of an internal standard (IS) mixture solution to facilitate quantification. Five isotopically labeled ISs were used for the quantification of the 17 targeted mycotoxins: U-[^13^C_34_]-FUMB_1_ for FUMB_1_ and FUMB_2_; U-[^13^C_20_]-OTA for OTA; U-[^13^C_18_]-ZEN for ZEN, ENB, ENB_1_, ENA, and ENA_1_; and U-[^13^C_15_]-DON for DON (Table 7).

### 4.4. Mycotoxin Analysis

Mycotoxin analysis was conducted using an LCMS-8040 system, comprising a UHPLC module coupled to a triple-quadrupole mass spectrometer, with data acquisition and processing performed via LabSolutions Software v5.91 (2017) from Shimadzu (Tokyo, Japan). For chromatographic separation, a Phenomenex C18 column (50 × 2.1 mm I.D., 100 Å) maintained at 50 °C was used, with a mobile phase of water/acetic acid (99.5:0.5, *v*:*v*; Phase A) and isopropanol/acetic acid (99.5:0.5, *v*:*v*; Phase B) delivered at 0.4 mL/min. The gradient program progressed as follows: 90% A at 0.01 min, 45% A at 1.5 min, 15% A at 3.5 min, 20% A at 4.0 min, and 98% A from 4.01 to 11.0 min, with a 50 μL injection volume. The mass spectrometer was operated in dual-polarity electrospray ionization (ESI±) mode with multiple reaction monitoring (MRM), tracking two transitions per analyte, quantification (Q) and qualification (q), under optimized parameters: desolvation line temperature: 250 °C; heater block: 400 °C; nebulizing gas (N_2_): 2.5 L/min; drying gas (N_2_): 15 L/min; and collision-induced dissociation gas (Ar) at 270 kPa. Analytical parameters are listed in Table 8.

**Figure 1:**
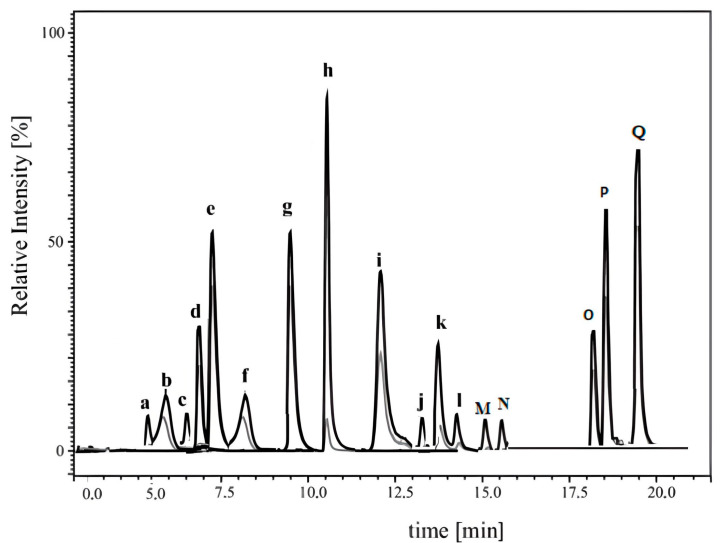
LC-MS/MS chromatogram of the 17 mycotoxins β-ZOL (a), α-ZON (b), HT2 (c), T2 (d), ZEN (e), NIV (f), CIT (g), OTA (h), ENA (i), ENB1 (j), ENA1 (k) FB1 (l), FB2 (M), FUS-X (N), AcDON (O), 3ADON (P), and DON (Q).

The methodology proposed for mycotoxin determination was optimized in terms of recoveries, linearity, and limits of detection (LOD) and quantification (LOQ). All calibration curves exhibited good linearity across the studied concentration ranges, with correlation coefficients (R^2^) exceeding 0.87 in all cases (Table 9). The limits of detection (LODs) and quantification (LOQs) ranged from 0.005 to 5 μg/kg and 0.015 to 15 μg/kg, respectively. Recovery rates for all analyzed mycotoxins were above 88%, indicating satisfactory method performance in accordance with the criteria established by the European Commission Decision 2002/657/EC [10]. The chromatogram of the analyzed mycotoxins is shown in Figure 1.

### 4.5. Risk Assessment Determination

#### 4.5.1. Dietary Exposure to Mycotoxins

To assess dietary exposure to mycotoxins among Tunisian adults, the Estimated Daily Intake (*EDI*) was calculated. The *EDI* (expressed in ng/kg body weight/day) was determined using the following formula [26]:

(1) $$EDI=\frac{MC\times ADC}{BW}$$

where *MC* is the mean mycotoxin concentration in the analyzed wheat samples (ng/kg); *ADC* is the average daily wheat consumption by the Tunisian population (g/day); and *BW* is the average body weight, standardized at 70 kg for Tunisian adults [27].

#### 4.5.2. Risk Characterization

For the risk characterization of OTA, a mycotoxin exhibiting both carcinogenic and genotoxic properties, the Margin of Exposure (MOE) approach was employed. This methodology was selected due to the absence of established toxicological thresholds (e.g., tolerable daily intake) for OTA, as noted by the EFSA [68]. For ZEN and DON, the Hazard Quotient (HQ) was applied, which compares estimated dietary exposure (EDI) levels to their respective health-based guidance values (HBGVs) derived from toxicological reference points. For ENs, quantitative risk characterization was not feasible owing to insufficient toxicological data.

##### Margin of Exposure (MOE) Determination

The MOE methodology serves as a critical quantitative risk assessment framework for evaluating health risks associated with chemical contaminants in food and feed [76]. This approach is specifically designed for substances exhibiting both genotoxic and carcinogenic properties, where conventional health-based guidance values cannot be reliably established. The MOE calculation integrates toxicological and exposure data through the following equation:

(2) $$MOE=\frac{{BMDL}_{10}}{EDI}$$

where BMDL is the Benchmark Dose Lower Limit which is a statistically determined lower confidence limit of the dose associated with a predefined toxicological effect level (causing no more than 10% of cancer incidence). It is derived through quantitative dose–response modeling of experimental or epidemiological data, serving as a critical reference point in toxicological risk assessment, expressed in ng/kg bw/day, and EDI is the Estimated Daily Intake, expressed in ng/kg bw/day.

The MOE was specifically calculated for OTA, and a BMDL_10_ of 14.5 μg/kg bw/day was used [68]. An MOE ≥ 10,000 indicates minimal risk, whereas an MOE < 10,000 indicates potential health issues necessitating immediate attention from risk managers [76].

##### Hazard Quotient (HQ) Determination

The Hazard Quotient (HQ) is a risk assessment ratio that compares estimated human exposure to a mycotoxin with a toxicological reference dose, such as the tolerable daily intake (TDI), at which no adverse effects are expected [77].

The HQ is calculated as follows:

(3) $$HQ=\frac{EDI}{TDI}$$

where EDI (ng/kg bw/day) refers to the Estimated Daily Intake and TDI (ng/kg bw/day) represents the tolerable daily intake value.

Based on its estrogenic effects observed in pigs, the EFSA [67] established a TDI of 250 ng/kg bw/day for ZEN. Additionally, the EFSA set a TDI of 1000 ng/kg bw/day for DON due to its impact on reducing body weight gain in mice. When the Hazard Quotient (HQ) is less than 1, it indicates a tolerable level of exposure. Conversely, an HQ greater than 1 suggests potential adverse health effects [77].

### 4.6. Statistical Study

Data were analyzed using the SPSS software program (SPSS Institute, Inc, Chicago, Ill, USA, 2000, Version 10.0), and comparisons of means between sampling periods were conducted using the ANOVA test (*p* < 0.05).

## Figures and Tables

**Table 1:** Occurrence and contamination levels of the detected mycotoxins in durum wheat samples from Tunisia.

Total Samples Collected	Detected Mycotoxin	Number of Positive Samples	Frequency (%)	Mean Concentration ^1^ (µg/Kg)	Range (µg/Kg)
167	ZEN	65	38.92	26.85	10.14–255.41
	DON	62	37.12	0.68	0.01–9.11
	OTA	68	40.71	1.85	1.02–11.45
	ENA1	10	5.98	1.69	2.10–70.5
	ENB	51	30.53	10.13	8.20–384.20
	ENB1	20	11.98	3.38	3.29–71.23

Only positive samples (>LOD) were considered for calculations. ^1^ Mean concentration in all samples.

**Table 2:** Moisture content and water activity levels in pre- and post-harvest wheat samples collected in Tunisia 2021 and 2022.

Agricultural Practices	Year	Phase	Water Activity (aw) Values	Moisture Content Values
NT	2021	Pre-harvest	0.59–0.69	11–14%
		Post-harvest	0.63–0.70	12–15%
CT	2022	Pre-harvest	0.61–0.68	13–16%
		Post-harvest	0.62–0.69	14–16%

NT: no-tillage practices; CT: conventional tillage practices.

**Table 3:** Mycotoxin prevalence in Tunisian durum wheat samples collected in 2021 under no-tillage (NT) practices.

Detected Mycotoxin	Pre-Harvest (n = 37)				Post-Harvest (n = 36)				Positive Samples/Total Samples (Frequency, %)
	Positive Samples	Frequency (%)	Mean ^1^ (µg/Kg)	Range (µg/Kg)	Positive Samples	Frequency (%)	Mean ^1^ (µg/Kg)	Range (µg/Kg)	
ZEN	22	59.45	26.91	10.14–210.11	23	63.88	63.78	12.11–255.41	45/73 (61.64)
DON	17	45.94	1.13	0.08–9.11	18	50	0.42	0.01–2.15	35/73 (47.94)
OTA	13	35.13	1.60	1.56–9.55	13	36.11	1.80	1.23–11.45	26/73 (35.61)
ENA1	2	5.40	0.43	5.52–10.74	1	2.77	0.40	14.55	3/73 (4.10)
ENB	17	45.94	19.26	8.81–247	3	3	1.12	12.50–14.22	20/73 (27.39)
ENB1	14	37.83	9.62	11.49–71.23	0	0	0	0	0

Only positive samples (>LOD) were considered for calculations. ^1^ Mean concentration of mycotoxin in all samples; n: total number of collected samples; NT: no-tillage practices.

**Table 4:** Mycotoxin prevalence in Tunisian durum wheat samples collected in 2022 under conventional tillage (CT) practices.

Detected Mycotoxin	Pre-Harvest (n = 49)				Post-Harvest (n = 45)				Positive Samples/Total Samples (Frequency, %)
	Positive Samples	Frequency (%)	Mean ^1^ (µg/Kg)	Range (µg/Kg)	Positive Samples	Frequency (%)	Mean ^1^ (µg/Kg)	Range (µg/Kg)	
ZEN	2	4.08	10.41	44.56–122.56	18	40	28.32	10.71–211.25	20/94 (21.27)
DON	6	12.24	0.64	0.12–7.85	21	46.66	0.84	0.05–5.14	27/94 (28.72)
OTA	20	40.81	1.67	1.02–8.56	22	48.88	2.27	2.33–9.78	42/94 (44.68)
ENA1	4	8.16	2.90	5.00–70.5	3	6.66	0.69	2.10–20.78	7/94 (7.44)
ENB	14	28.57	8.51	9.12–163.2	17	37.77	7.33	8.20–63.3	31/94 (32.98)
ENB1	8	16.32	3.23	6.70–54	12	26.66	4.82	3.29–60.42	20/94 (21.27)

^1^ Mean concentration of mycotoxin in all samples; n: total number of collected samples; CT: conventional tillage practices.

**Table 5:** Co-occurrence of mycotoxins in Tunisian durum wheat samples collected in 2021 and 2022.

Year (Farmers’ Practice Type)		Contamination (Number of Positive Samples, Frequency)		
	by 2 Mycotoxins	by 3 Mycotoxins	by 4 Mycotoxins	by 5 Mycotoxins
2021 (NT) N = 73	ZEN + OTA DON + OTA ZEN + DON DON + ENB DON + ENB1 (20, 27%)	ZEN + ENB + ENB1 ZEN + OTA + ENB1 ZEN + OTA + DON (7, 10%)	DON + ENA1 + ENB + ENB1 ZEN + OTA + ENB + ENB1 ZEN + DON + ENA1 + ENB (5, 7%)	ZEN + DON + OTA + ENB + ENB1 ZEN + DON + ENA1 + ENB + ENB1 (4, 5%)
2022 (CT) N = 94	ENB + ENB1 ZEN + OTA ZEN + DON ZEN + ENB1 DON + OTA OTA + ENB OTA + ENB1 OTA + ENA1 DON + ENB (26, 28%)	ZEN + OTA + DON OTA + ENB + ENB1 ZEN + ENB + ENB1 DON + ENB + ENB1 DON + OTA + ENB (15, 16%)	DON + ENA1 + ENB + ENB1 DON + OTA + ENB + ENB1 DON + ENA1 + ENB1 (5, 5%)	ZEN + OTA + ENA1 + ENB + ENB1 ZEN + OTA + ENA1 + ENB + ENB1 ZEN + DON + OTA + ENA1 + ENB1 (3, 3%)
Total (n = 167)	(46, 28%)	(22, 13%)	(10, 6%)	(7, 4%)

n: total number of collected samples; NT: no-tillage practices; CT: conventional tillage practices.

**Table 6:** Estimated Daily Intake (EDI) of detected mycotoxins and risk characterization components (Margin of Exposure (MOE), Hazard Quotient (HQ)) in durum wheat samples collected in Tunisia in 2021 and 2022.

Detected Mycotoxin	Mean Concentration ^1^ (µg/Kg)	EDI (ng/Kg bw/day)	HQ	MOE
**2021 (NT)**				
OTA	1.69	17.29	-	838.63
ZEN	45.58	466.31	1.87	
DON	0.78	8.00	0.008	
ENA1	0.42	4.30	-	-
ENB	10.28	105.17	-	-
ENB1	4.88	49.91	-	-
**2022 (CT)**				
OTA	1.96	20.08	-	722.11
ZEN	18.99	194.74	0.80	-
DON	0.73	7.56	0.007	
ENA1	1.84	18.85	-	-
ENB	11.82	121.08	-	-
ENB1	4.00	40.92	-	-

^1^ Mean concentration of mycotoxin in all samples; NT: no-tillage practices; CT: conventional tillage practices.

**Table 7:** UHPLC-MS/MS parameters for internal standards used in mycotoxin determination.

Internal Standards	Polarity	MRM Q	EC MRM Q
U-(13C34)-FUMB1	+	756.300 > 356.500	−44
U-(13C20)-OTA	+	424.600 > 250.250	−21
U-(13C18)-ZEN	−	331.100 > 185.000	28
U-[13C15)-DON	−	370.800 > 59.000	24

MRM Q: multiple reaction monitoring, quantitative; EC MRM Q: energy collision MRM quantification.

**Table 8:** UHPLC-MS/MS analytical parameters for multi-mycotoxin determination.

Mycotoxins	MRM Q	EC MRM Q	Polarity	MRM QUAL	EC MRM q
OTA	404.0 > 238.9	−26	+	404.0 > 358.0	−16
ZEN	317.1 > 175.2	25	−	317.1 > 131.0	30
DON	355.1 > 59.0	35	−	355.1 > 265.1	35
3ADON	397.2 > 59.0	25	−	397.2 > 307.1	16
15ADON	338.9 > 137.1	−19	+	338.9 > 297.2	−14
FUMB1	722.3 > 334.4	−43	+	722.3 > 352.4	−44
FUMB2	706.2 > 318.4	−37	+	706.2 > 336.4	−44
FUS-X	355.0 > 175.10	−19	+	355.0 > 247.1	−12
ENA	682.5 > 210.0	−25	+	682.5 > 228.0	−39
ENA1	668.5 > 100.1	−30	+	668.5 > 210.0	−12
ENB	640.4 > 196.0	−24	+	640.4 > 520.6	−40
ENB1	654.50 > 196.0	−25	+	654.5 > 57.1	−36
CIT	355.0 > 175.10	−24	+	355.0 > 247.1	−24
NEO	400.2 > 185.1	−22	+	400.2 > 215.1	−16
HT2	447.2 > 345.2	−18	+	447.2 > 285.1	−21
T2	484.0 > 215.1	−19	+	484.0 > 305.2	−14
NIV	371.0 > 281.1	17	−	371.0 > 311.2	12

MRM Q: multiple reaction monitoring, quantitative; EC MRM Q: energy collision MRM quantification; MRM qual: MRM, qualitative; EC MRM q: EC MRM, qualitative.

**Table 9:** Analytical performance parameters (LOD, LOQ, R^2^) for mycotoxin determination.

Mycotoxin	LOD (µg/kg)	LOQ (µg/kg)	R2
OTA	0.3	0.9	0.8750
ZEN	0.5	1.5	0.9733
DON	0.005	0.015	0.9506
3ADON	0.005	0.015	0.9901
15ADON	0.005	0.015	0.9900
FB1	1.0	3	0.9996
FB2	3	9	0.9980
FUS-X	5	15	0.9967
ENA	0.3	0.9	0.9874
ENA1	0.2	0.6	0.9928
ENB	0.5	1.5	0.9921
ENB1	0.5	1.5	0.9975
CIT	0.5	1.5	0.9988
NEO	5	15	0.9967
HT2	5	15	0.9961
T2	5	15	0.9904
NIV	5	15	0.9992

LOD: limit of detection; LOQ: limit of quantification; R^2^: correlation coefficients.

## Data Availability

The original contributions presented in this study are included in the article. Further inquiries can be directed to the corresponding authors.
